# Supplementary material for: Admixture mapping of peripheral artery disease in a Dominican population reveals a putative risk locus on 2q35
Source: Front Genet. 2023 Aug 1;14:1181167. doi: 10.3389/fgene.2023.1181167 (PMC10432698; doi:10.3389/fgene.2023.1181167)
Supplement: Supplementary file 1 [file DataSheet1.pdf]

Supplementary Material

1.1 Supplementary Figures

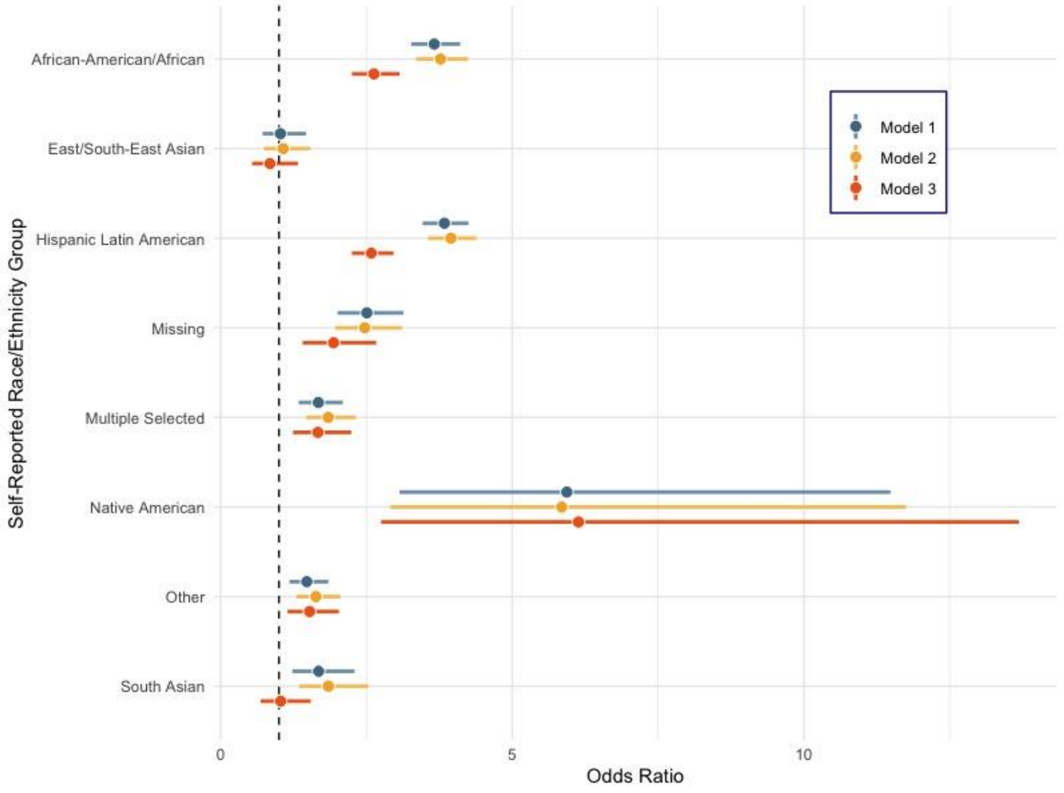

Supplementary Figure 1. *Odds of peripheral artery disease (PAD) across seven self-report race/ethnicity groups, and one group with missing data, in BioMe compared to the self-reported European population. Model 1: PAD ~ Population group + Age + Sex, Model 2: PAD ~ Model 1 + BMI, Model 3: PAD ~ Model 2 + T2D + TG + TC + HDL.*

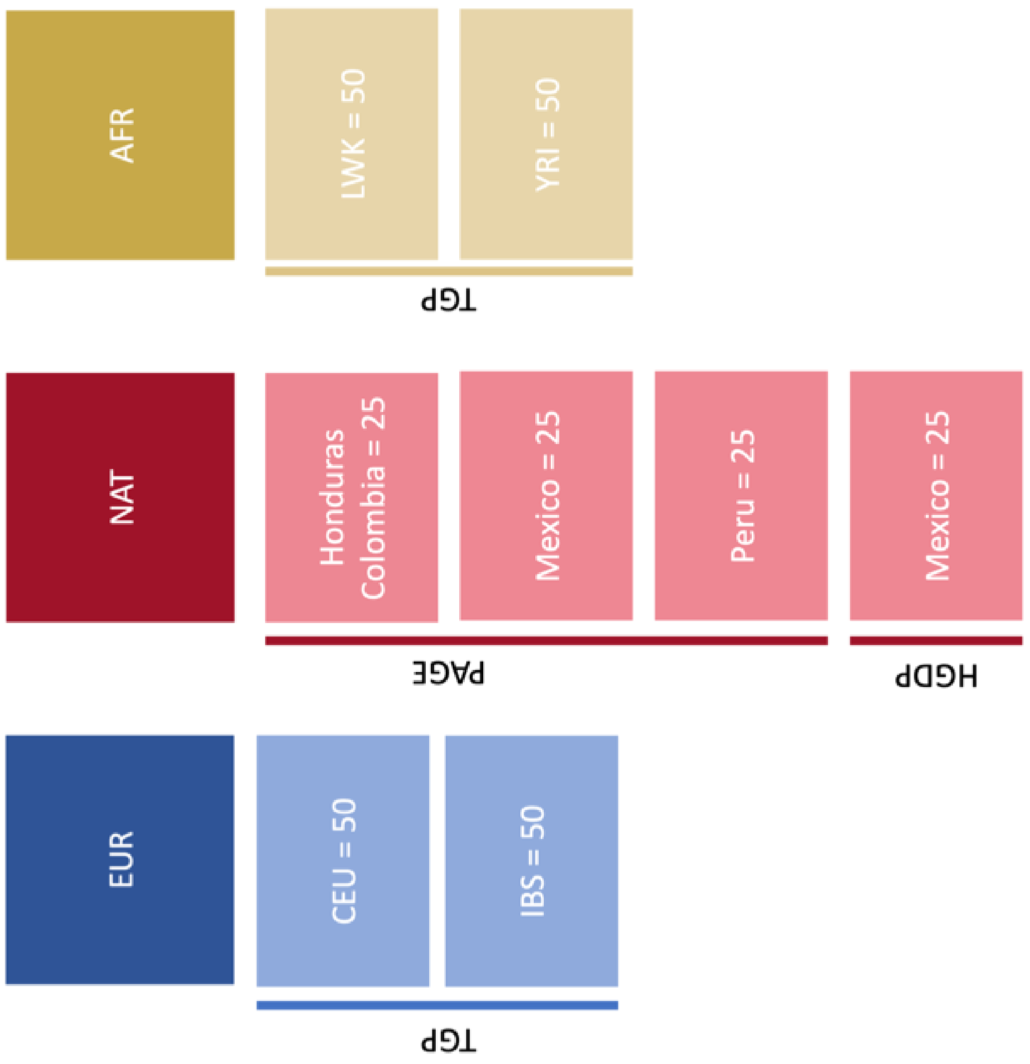

Supplementary Figure 2. *Schema of reference panel used for local ancestry inference. This reference panel is also included in the admixture plot (Supplemental Figure 3A) for interpretation purposes.*

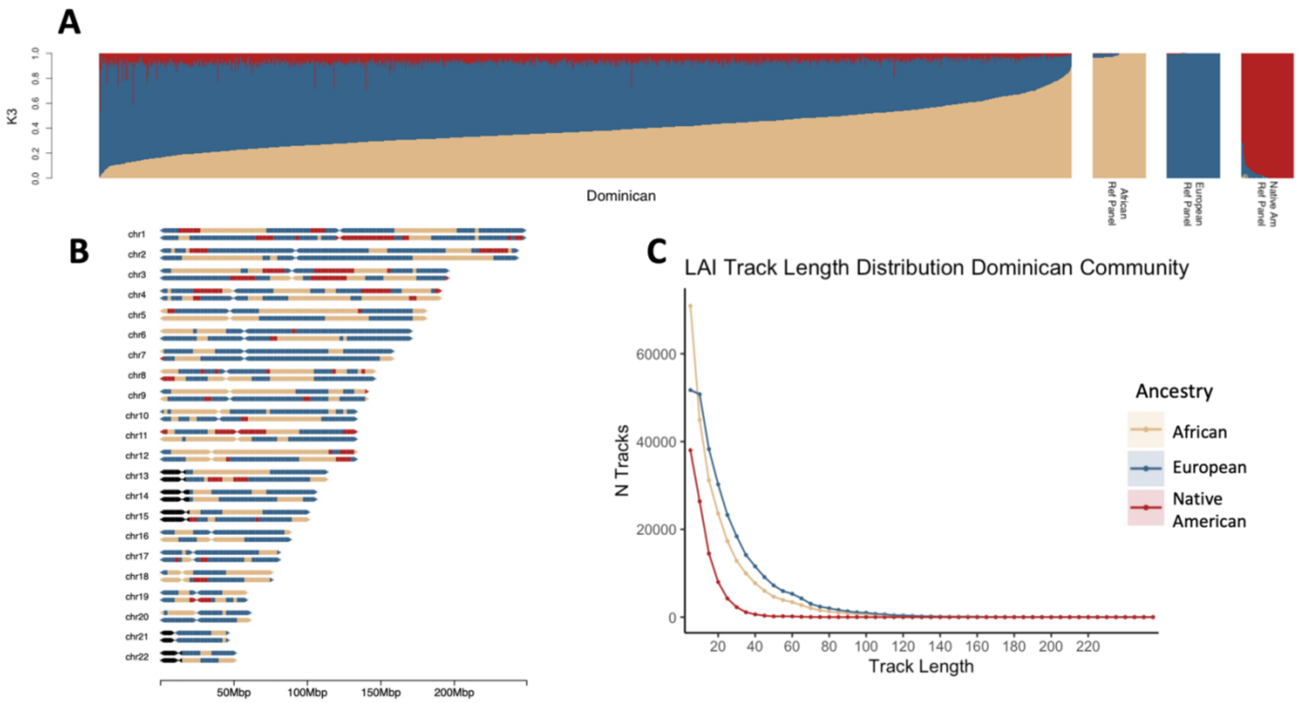

Supplementary Figure 3. ***A)*** *Admixture plots of individuals that are members of the Dominican IBD community in the BioMe biobank. Thousand Genomes Project (TGP), Human Genome Diversity Project (HGDP) and PAGE Native American samples were used to make the reference panels included.* ***B)*** *Karyogram plot with colors representing local ancestry tracts for a Dominican individual with approximately 57% European (EUR), 37% African (AFR) and 6% Native American (NAT) genetic ancestry.* ***C)*** *Tract length distribution of local ancestry haplotypes.*

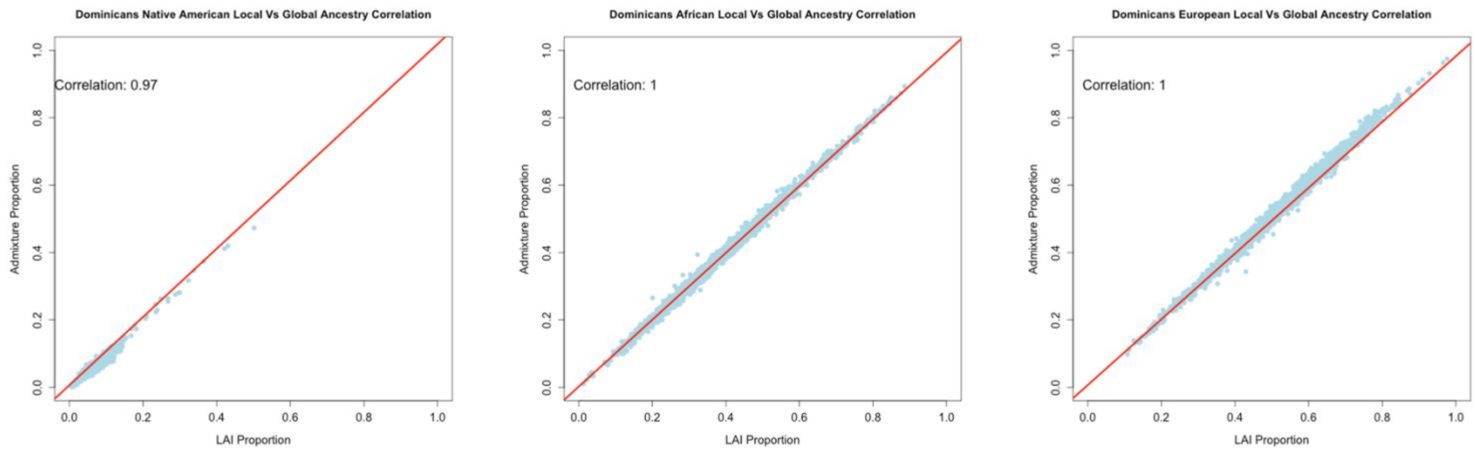

Supplementary Figure 4. *Quality control of local ancestry calls. Local ancestry calls are summed per individual and compared to global ancestry proportions calculated using ADMIXTURE software.*

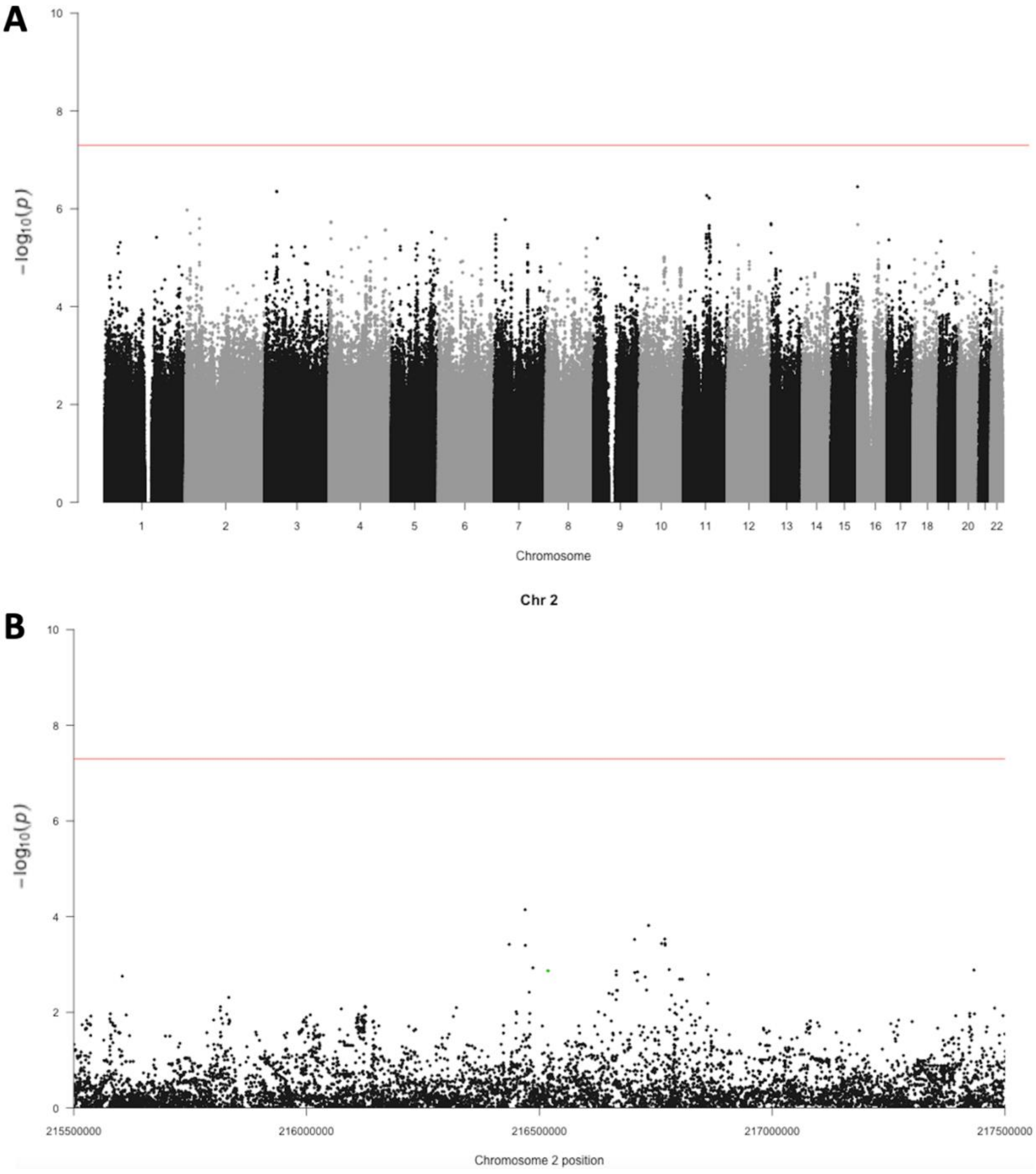

Supplementary Figure 5. ***A)*** *Manhattan plot showing -log*$_{10}$ *p-values from PAD GWAS meta-analysis in the BioMe Dominican discovery cohort using imputed MEGA and OMNI genotype data. Genome-wide significance threshold shown in red.* ***B)*** *Zoom of Manhattan plot A highlighting 2q35 region, tag SNP rs78529201 is shown in green.*

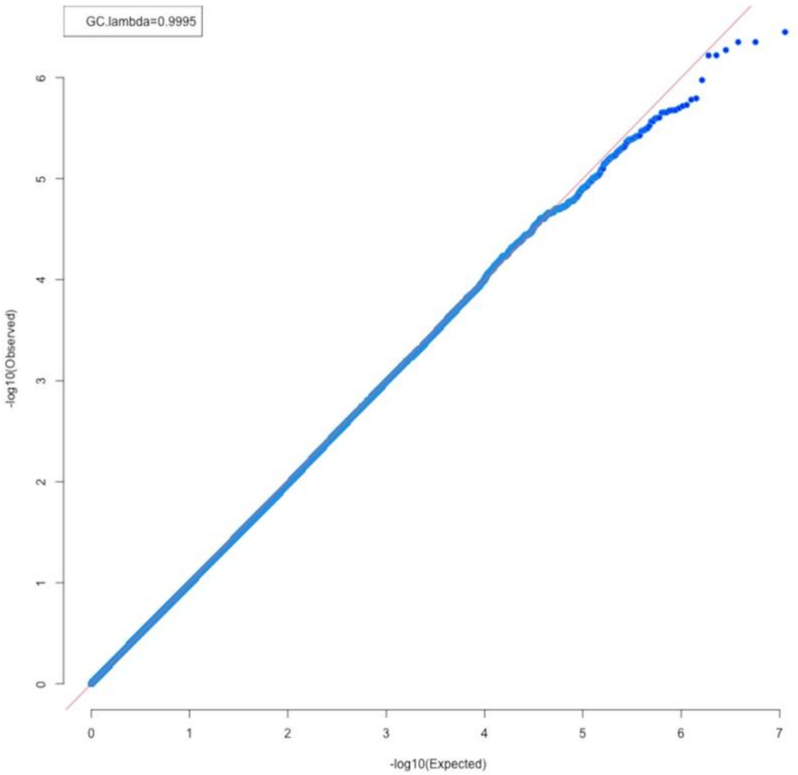

Supplementary Figure 6. *Quantile-quantile (QQ) plot of -log$_{10}$ p-values from PAD GWAS meta-analysis in the BioMe Dominican discovery cohort using imputed MEGA and OMNI genotype data.*

1.2 Supplementary Tables

	Sample Size	N Female (%)	N PAD Cases (%)	Median Age(SD)	Genotyping Panels
Dominicans (Admixture Mapping)	1813	1184 (65.3%)	245 (13.5%)	56 (16.62)	MEGA and OMNI
Dominicans (GWAS)	803	525 (65.4%)	121(15%)	58 (14.9)	MEGA
	862	558 (64.7%)	118 (13.7%)	55 (17.1)	OMNI

Supplementary Table 1. *Summary of Dominican cohort used in discovery admixture mapping analysis and GWAS.*

	Model 1			Model 2			Model 3		
Race/Ethnicity	P-value	Odds Ratio (95% CI)	Sample Size	P-value	Odds Ratio (95% CI)	Sample Size	P-value	Odds Ratio (95% CI)	Sample Size
African-American/African	1.58E-109	3.66 (3.27-4.10)	11462	3.57E-109	3.77 (3.35-4.24)	10796	2.26E-34	2.63 (2.25-3.07)	6512
East/South-East Asian	8.89E-01	1.02 (0.72-1.46)	2044	7.11E-01	1.07 (0.75-1.54)	1842	4.66E-01	0.85 (0.54-1.33)	1026
Hispanic Latin American	2.77E-146	3.84 (3.46-4.25)	19547	1.37E-145	3.95 (3.56-4.39)	18400	1.02E-41	2.58 (2.25-2.97)	10656
Missing	7.40E-16	2.51 (2.00-3.13)	2087	1.22E-14	2.47 (1.96-3.11)	1963	5.41E-05	1.94 (1.4-2.67)	877
Multiple Selected	6.57E-06	1.67 (1.34-2.1)	2191	1.59E-07	1.84 (1.47-2.32)	1933	6.57E-04	1.67 (1.24-2.24)	944
Native American	1.23E-07	5.93 (3.07-11.49)	80	7.03E-07	5.85 (2.91-11.75)	73	9.33E-06	6.14 (2.75-13.69)	45
Other	6.50E-04	1.48 (1.18-1.85)	1905	2.60E-05	1.63 (1.3-2.05)	1696	3.68E-03	1.53 (1.15-2.03)	925
South Asian	1.12E-03	1.68 (1.23-2.3)	1478	1.36E-04	1.85 (1.35-2.53)	1333	8.87E-01	1.03 (0.69-1.55)	727

Supplementary Table 2. *Results of peripheral artery disease (PAD) risk analysis across self-reported groups in BioMe compared to the self-reported European population. Model 1: PAD ~ Population group + Age + Sex , Model 2: PAD ~ Model 1 + BMI, Model 3: PAD ~ Model 2 + T2D + TG + TC + HDL.*

	Model 1			Model 2			Model 3		
Race/Ethnicity	P-value	Odds Ratio (95% CI)	Sample Size	P-value	Odds Ratio (95% CI)	Sample Size	P-value	Odds Ratio (95% CI)	Sample Size
African-American/African	1.01E-47	3.58 (3.01-4.25)	7127	5.23E-44	3.49 (2.92-4.16)	6642	8.05E-13	2.31 (1.84-2.9)	4256
Ashkenazi Jewish	7.47E-01	0.96 (0.77-1.2)	4236	2.91E-01	0.88 (0.7-1.11)	3775	0.32	0.87 (0.65-1.15)	1965
Dominican	1.56E-39	4.12 (3.34-5.09)	1939	3.91E-36	4.05 (3.26-5.05)	1763	6.44E-14	3.15 (2.33-4.25)	980
Ecuadorian	1.44E-05	2.46 (1.64-3.7)	432	4.05E-05	2.38 (1.57-3.61)	399	0.054	1.67 (0.99-2.82)	236
Filipino and other Southeast Asian	6.45E-01	1.15 (0.64-2.06)	602	7.93E-01	1.08 (0.59-1.99)	526	0.49	0.76 (0.35-1.64)	312
Other Central and South American	2.56E-09	2.48 (1.84-3.34)	1012	1.67E-08	2.42 (1.78-3.29)	934	4.53E-04	2.01 (1.36-2.96)	541
Puerto Rican	3.89E-61	4.26 (3.59-5.06)	5292	1.91E-55	4.09 (3.43-4.88)	4941	2.61E-12	2.27 (1.8-2.86)	3243

Supplementary Table 3. *Results of peripheral artery disease (PAD) risk analysis across diverse genetic ancestry groups in BioMe compared to the non-Jewish European population. Model 1: PAD ~ Population group + Age + Sex, Model 2: PAD ~ Model 1 + BMI, Model 3: PAD ~ Model 2 + T2D + TG + TC + HDL.*
